# Draft Genome Sequences of Two Strains of a Newly Described Species, *Sphingobacterium cellulitidis*

**DOI:** 10.1128/genomeA.00956-17

**Published:** 2017-09-21

**Authors:** Torsten Seemann, Dieter M. Bulach, Glen Carter, M. John Albert

**Affiliations:** a Melbourne Bioinformatics, The University of Melbourne, Carlton, Australia; b Doherty Applied Microbial Genomics, Department of Microbiology and Immunology, The University of Melbourne at The Peter Doherty Institute for Infection & Immunity, Melbourne, Australia; c Department of Microbiology, Faculty of Medicine, Kuwait University, Jabriya, Kuwait

## Abstract

The draft genome sequences of two strains of a newly described species,* Sphingobacterium cellulitidis*, have been determined. The type strain originated from cellulitis of a toe of a patient and the other strain from the environment. The sequences will provide the reference genomes of the new *Sphingobacterium* species.

## GENOME ANNOUNCEMENT

The genus *Sphingobacterium* comprises Gram-negative, nonfermenting, non-spore-forming aerobic bacilli. These bacteria are catalase and oxidase positive. They can be cultivated on blood agar and MacConkey agar, where they produce colonies with a yellow color (1). They are ubiquitous in nature, being found in soil, plant material, and water bodies. They are characterized by the presence of menaquinone as the major respiratory quinone, as well as sphingolipids and polar lipids (2). There are 43 species of *Sphingobacterium*, and few are associated with human infection, with the exception of *S. spiritivorum* and *S. multivorum*. These two species are occasionally isolated from bloodstream infections, respiratory infections, cellulitis, and end-stage kidney disease (3–8). *Sphingobacterium* strain R-53603 was isolated from cellulitis of the right big toe of a female patient in Kuwait, and according to 16S rRNA gene sequence analysis, this strain is closely related to environmental strain R-53745 collected from an activated sludge isolate in Singapore. DNA-DNA hybridization and other analyses were used to provide evidence leading to the assignment of these strains to a new species, *Sphingobacterium cellulitidis* (9). Reported here are the annotated draft genome sequences for both R-53603 (the type strain for *S. cellulitidis*) and R-53745. Significantly, this is a third *Sphingobacterium* species associated with human disease.

Whole-genome sequencing of the strains was performed on an Illumina NextSeq 500 instrument using Nextera XT library preparation and paired-end 151-bp chemistry. Sequence reads were *de novo* assembled using Unicycler version 0.4 (10), with default parameters, and annotated using Prokka version 1.12 (11), with default parameters.

The characteristics of genome assembly and annotation of strains R-53603 and R-53745 are as follows: sequencing depths, 94× and 106×; sizes, 4.61 Mb and 4.40 Mb; numbers of contigs (≥200 bp), 32 and 51; contig *N*_50_ values, 438 kbp and 225 kbp; G+C contents, 37.5% and 37.4%; numbers of coding sequences (CDSs), 4,011 and 3,771; and numbers of tRNAs, 45 and 45, respectively. No antibiotic resistance genes (http://cge.cbs.dtu.dk/services/ResFinder/) or virulence factor genes (http://www.mgc.ac.cn/VFs/) were detected in the genome sequence of either *S. cellulitidis* strain.

### Accession number(s).

This whole-genome shotgun project has been deposited in DDBJ/ENA/GenBank under the accession numbers NOUX00000000 (strain R-53603) and NOUY00000000 (strain R-53745). The versions described in this paper are the first versions, NOUX01000000 and NOUY01000000, respectively.

## References

[1] Forbes B, Sahm D, Weissfeld A 2007 Diagnostic microbiology, 12th ed. Elsevier, Philadelphia, PA.

[2] Wauters G, Janssens M, De Baere T, Vaneechoutte M, Deschaght P 2012 Isolates belonging to CDC group II-i belong predominantly to *Sphingobacterium mizutaii* Yabuuchi et al. 1983: emended descriptions of *S. mizutaii* and of the genus *Sphingobacterium*. Int J Syst Evol Microbiol 62:2598–2601. doi:10.1099/ijs.0.037325-0. 22199213

[3] Barahona F, Slim J 2015 *Sphingobacterium multivorum*: case report and literature review. New Microbes New Infect 7:33–36. doi:10.1016/j.nmni.2015.04.006. 26236492 PMC4501434

[4] Marinella MA 2002 Cellulitis and sepsis due to *Sphingobacterium*. JAMA 288:1985. 12387649 10.1001/jama.288.16.1985-a

[5] Tronel H, Plesiat P, Ageron E, Grimont PA 2003 Bacteremia caused by a novel species of *Sphingobacterium*. Clin Microbiol Infect 9:1242–1244. doi:10.1111/j.1469-0691.2003.00801.x. 14686992

[6] Kämpfer P, Engelhart S, Rolke M, Sennekamp J 2005 Extrinsic allergic alveolitis (hypersensitivity pneumonitis) caused by *Sphingobacterium spiritivorum* from the water reservoir of a steam iron. J Clin Microbiol 43:4908–4910. doi:10.1128/JCM.43.9.4908-4910.2005. 16145174 PMC1234114

[7] Gupta A, Logan J, Elhag N, Almond M 2016 *Sphingobacterium spiritivorum* infection in a patient with end stage renal disease on haemodialysis. Ann Clin Microbiol Antimicrob 15:25. doi:10.1186/s12941-016-0141-5. 27090094 PMC4835858

[8] Lambiase A, Rossano F, Del Pezzo M, Raia V, Sepe A, de Gregorio F, Catania MR 2009 *Sphingobacterium* respiratory tract infection in patients with cystic fibrosis. BMC Res Notes 2:262. doi:10.1186/1756-0500-2-262. 20030840 PMC2805677

[9] Huys G, Purohit P, Tan CH, Snauwaert C, De Vos P, Saffar HA, Al Obaid I, Busse HJ, Seemann T, Albert MJ 2017 *Sphingobacterium cellulitidis* sp. nov., isolated from clinical and environmental sources. Int J Syst Evol Microbiol 67:1415–1421. doi:10.1099/ijsem.0.001832. 28141505

[10] Wick RR, Judd LM, Gorrie CL, Holt KE 2017 Unicycler: resolving bacterial genome assemblies from short and long sequencing reads. PLoS Comput Biol 13:e1005595. doi:10.1371/journal.pcbi.1005595. 28594827 PMC5481147

[11] Seemann T 2014 Prokka: rapid prokaryotic genome annotation. Bioinformatics 30:2068–2069. doi:10.1093/bioinformatics/btu153. 24642063

